# Associations of second-hand smoke exposure with hand eczema and atopic dermatitis among college students in China

**DOI:** 10.1038/s41598-020-74501-2

**Published:** 2020-10-15

**Authors:** Danrong Jing, Juan Li, Juan Tao, Xiaohui Wang, Shijun Shan, Xiaojing Kang, Bin Wu, Yichi Zhang, Yi Xiao, Xiang Chen, Minxue Shen

**Affiliations:** 1 Department of Dermatology, Xiangya Hospital, Central South University, 87 Xiangya Road, Changsha, 410008 Hunan China; 2 Institute of Clinical Pharmacology, Xiangya Hospital, Central South University, Changsha, China; 3 Department of Dermatology, Union Hospital, Tongji Medical College, Huazhong University of Science and Technology, Wuhan, China; 4 Department of Dermatology, Zhongshan Hospital, Xiamen University, Xiamen, China; 5 Department of Dermatology, Xiang’an Hospital, Xiamen University, Xiamen, China; 6 Department of Dermatology, People’s Hospital of Xinjiang Uygur Autonomous Region, Urumqi, Xinjiang China; 7 Department of Dermatology, The Affiliated People’s Hospital of Inner Mongolia Medical University, Hohhot, China; 8 The First High School of Changsha, Changsha, China; 9 Hunan Engineering Research Center of Skin Health and Disease, Central South University, Changsha, China; 10 Hunan Key Laboratory of Skin Cancer and Psoriasis, Central South University, Changsha, China; 11 National Clinical Research Center for Geriatric Disorders, Xiangya School of Public Health, Central South University, Changsha, China; 12 Department of Social Medicine and Health Management, Xiangya School of Public Health, Central South University, Changsha, China

**Keywords:** Skin diseases, Environmental impact

## Abstract

Smoking has been identified as a risk factor for atopic dermatitis and hand eczema, but less is known about the association of exposure to second-hand smoke (SHS) with hand eczema. The study aimed to investigate the association of SHS exposure with hand eczema and atopic dermatitis in a group of adolescents. We conducted a cross-sectional study among first-year college students. SHS exposure was measured by a self-administered questionnaire. Skin diseases were diagnosed by dermatologists in the field survey. Mixed models were used to estimate the associations. A total of 20,129 participants that underwent skin examination and a questionnaire survey were included in the analyses. The prevalence rates of atopic dermatitis and hand eczema were 3.86% and 3.35%, respectively. Crude and adjusted estimates consistently showed that exposure to SHS was significantly associated with atopic dermatitis and hand eczema in a dose–response manner. Attention deficit/hyperactivity disorder mediated minimal or no effect of SHS on hand eczema and atopic dermatitis. Subgroup analysis by type of hand eczema, and sensitivity analysis by excluding data with center effect showed consistent results. Exposure to SHS is an independent but modifiable risk factor for hand eczema and atopic dermatitis in adolescents.

## Introduction

Tobacco smoking has been the major cause of preventable morbidity and mortality in the world. According to the World Health Organization’s recent report on global tobacco use, the Western Pacific Region, including China, is projected to overtake South East Asia as the region with the highest rate of smoking among men^1^. Exposure to second-hand smoke (SHS) is associated more than 600,000 deaths of non-smokers each year, and contributes to 1% of the total global disease burden, representing about 10–15% of the disease burden caused by active smoking^2^. In China, except public transport, no smoke-free legislation has been enacted for public environments such as universities, hospitals, restaurants, and government facilities^3^.

Atopic dermatitis and hand eczema are common skin diseases that have adverse consequences such as job loss and impaired quality of life^4,5^. Previous studies demonstrated that US and Danish adults with atopic dermatitis had higher risk for cardiovascular disease, and the association might be attributable to or mediated by poor health behaviors including smoking^6–8^. Systematic reviews suggest that active smoking is associated with increased risks of atopic dermatitis^9^ and hand eczema^10^, although many of the evidences were based on self report of disease. In contrast, the association of SHS exposure with atopic dermatitis remains inconsistent, and the magnitude of association is variegated across age group, geographic region, setting, study design, and sample size. Visually no study evaluated the association of SHS exposure with hand eczema. In the current study, we investigated the associations of SHS exposure with diagnosed atopic dermatitis and hand eczema in a group of homogeneous adolescents who have experienced comparable social and environmental transitions China.

## Methods

### Study design

This cross-sectional study was carried out in five universities in different regions of China during September to October, 2018. The first-year college students that consented to participate underwent skin health examinations as well as an online questionnaire survey immediately after their enrolment to the universities. The questionnaire survey was organized by the departments of student affairs of the universities. Details about the field survey methodologies and technologies could be found in previously published papers^11,12^. This study was approved by the medical ethics committee of Xiangya Hospital, Central South University (approval number: 201709993).

### Disease diagnosis

Diagnosis of skin diseases and inquiry of disease history were performed by certificated dermatologists during the skin health examination. Clinical manifestation, disease history, and family history were inquired, and physical examinations were conducted to diagnose skin diseases. Atopic Dermatitis was diagnosed according to The International Study of Asthma and Allergies in Childhood criteria (ISAAC criteria)^13^. Hand eczema was further classified according to the guideline from the Danish Contact Dermatitis Group^14^.

### Exposure measurement

The frequency and duration of SHS exposure were measured by a self-administered questionnaire. SHS exposure was assessed with the question “During the past month, on how many days per week have people smoked in your presence in any place?” The frequency of exposure during the past year was categorized into three groups: 0 day/week, 1 day/week, and ≥ 2 day/week. Cumulative SHS exposure was assessed with the question “How many years have you been exposed to second-hand smoke for at least one day per week” The duration of exposure was also categorized into three groups: < 2 years, 2–5 years, and ≥ 6 years.

### Covariates

The cluster-level potential cofounder was university (random effect). Individual-level potential cofounders included geographic region of hometown, demographic characteristics (age, gender, ethnicity), socioeconomic status (annual family income), family structure, body mass index (BMI), health-related behaviors (active smoking, alcohol drinking, and physical activity), attention deficit/hyperactivity disorder (ADHD), and self-reported asthma and allergic rhinitis. BMI was calculated as weight (kg)/height^2^ (m^2^); height and weight were measured by research nurse using standardized methods during the health examination. ADHD was tested as a mediator because it links both SHS and eczema^15,16^. ADHD was determined by the Chinese version of the ADHD Self-report Scale with a cutoff value of 17^17^.

### Statistical analyses

Continuous data are presented as the mean ± standard deviation, and between-group differences were tested using analysis of variance (ANOVA). Categorical data are presented as number (%), and between-group differences were tested using the chi-square test.

Considering potential center effect, mixed models (student as level-1 unit and university as level-2 unit) with logit link function for binary outcomes were used to estimate the effects of SHS exposure on atopic dermatitis and hand eczema, adjusting for level-1 and level-2 confounders. Null model (without independent variables) was used to detect center effect at the university level, and the intra-cluster correlation coefficients (ICCs) were reported to describe the center effect. Odds ratios (ORs) and adjusted odds ratios (AORs) with 95% confidence intervals (CIs) were used to demonstrate effect size. Adjustments for made for covariates including demographic characteristics (hometown region, age, gender, ethnicity), family characteristics (annual income and family structure), behavior factors (active smoking, alcohol drinking, physical activity), ADHD, asthma, and allergic rhinitis which were significantly associated with SHS exposure. We also examined the joint effect of frequency and duration of SHS exposure (cumulative effect) by deriving a combined variable with nine groups.

Mediation effect analysis for ADHD was conducted with the quasi-Bayesian Monte Carlo method^18^. Subgroup analysis was performed by subclasses of hand eczema. Sensitivity analysis was conducted by excluding the data from study sites which demonstrated significant center effect. Analyses were performed using R Statistical Software. The significance level was 0.05 for all hypothesis tests.

## Results

A total of 21,088 students from five universities consented to participate, underwent the skin health examination, and completed the online questionnaire survey. A total of 20,129 subjects with complete information were analyzed. The geographic distributions of the selected universities and the students’ hometown provinces are shown in Supplementary Fig. S1. The characteristics (age and gender) of the participants in the final analysis were not statistically different from the rest of the subjects. The characteristics of the participants stratified by the frequency and duration of SHS exposure are shown in Table 1. The mean age was 18.3 years with a small standard deviation, and 51.1% of the participants were male. SHS exposure showed a positive association with family income, ADHD, asthma, and allergic rhinitis.

**Table 1:** Characteristics of participants by the frequency and duration of second-hand smoke exposure.

Variable	Category	Total	Frequency of smoke exposure (day/week)			Duration of smoke exposure (years)		
			0	1	≥ 2	< 2	2–5	≥ 6
		N (%)	N (%)	N (%)	N (%)	N (%)	N (%)	N (%)
Study site	Changsha	5017	3817 (76.1)	783 (15.6)	417 (8.3)	3683 (73.4)	376 (7.5)	958 (19.1)
	Wuhan	5602	4452 (79.5)	820 (14.6)	330 (5.9)	4350 (77.6)	430 (7.7)	822 (14.7)
	Xiamen	4206	3224 (76.7)	694 (16.5)	288 (6.8)	3100 (73.7)	300 (7.1)	806 (19.2)
	Urumqi	2922	2421 (82.9)	261 (8.9)	240 (8.2)	2522 (86.3)	155 (5.3)	245 (8.4)
	Hohhot	2382	1971(82.7)	226 (9.5)	185 (7.8)	1890 (79.3)	142 (6.0)	350 (14.7)
Students’ hometown region	North	3605	2962 (82.2)	397 (11.0)	246 (6.8)	2785 (77.3)	229 (6.3)	591 (16.4)
	Northeast	631	524 (83.0)	76 (12.0)	31 (5.0)	476 (75.4)	44 (7.0)	111 (17.6)
	East	4466	3431 (76.8)	723 (16.2)	312 (7.0)	3455 (77.4)	281 (6.3)	730 (16.3)
	Central	4244	3265 (76.9)	673 (15.9)	306 (7.2)	3238 (76.3)	337 (7.9)	669 (15.8)
	South	1395	1005 (72.0)	245 (17.6)	145 (10.4)	1013 (72.6)	112 (8.0)	270 (19.4)
	Southwest	1802	1398 (77.6)	274 (15.2)	130 (7.2)	1295 (71.9)	164 (9.1)	343 (10.9)
	West	3986	3300 (82.8)	396 (9.9)	290 (7.3)	3283 (82.4)	236 (5.9)	467 (11.7)
Age (years)		18.3 ± 0.8	18.3 ± 0.8	18.2 ± 0.7	18.3 ± 0.7	18.3 ± 0.8	18.2 ± 0.7	18.2 ± 0.7
Body mass index (kg/m^2^)		21.3 ± 3.5	21.2 ± 3.4	21.4 ± 3.6	21.7 ± 3.8	21.3 ± 3.5	21.5 ± 3.6	21.5 ± 3.6
Gender	Male	10,283	7816 (76.0)	1526 (14.8)	941 (9.2)	7825 (76.1)	858 (8.3)	1600 (15.6)
	Female	9846	8069 (81.9)	1258 (12.8)	519 (5.3)	7720 (78.4)	545 (5.5)	1581 (16.1)
Ethnicity	Han	16,222	12,566 (77.5)	2414 (14.9)	1242 (85.1)	12,263 (75.6)	1208 (7.4)	2751 (17.0)
	Other	3907	3319 (85.0)	370 (9.5)	218 (5.6)	3282 (84.0)	195 (5.0)	430 (11.0)
Annual family income (CNY)	< 10,000	2169	1828 (84.3)	219 (10.1)	122 (5.6)	1796 (82.8)	123 (5.7)	250 (11.5)
	10,001 to 30,000	4377	3541 (80.9)	564 (12.9)	272 (6.2)	3403 (77.7)	319 (7.3)	655 (15.0)
	30,001 to 50,000	3468	2754 (79.4)	464 (13.4)	250 (7.2)	2636 (76.0)	282 (8.1)	550 (15.9)
	50,001 to 99,999	4416	3409 (77.2)	660 (15.0)	347 (7.9)	3336 (75.5)	317 (7.2)	763 (17.3)
	100,000 to 199,999	4063	3126 (76.9)	620 (15.3)	317 (7.8)	3128 (77.0)	269 (6.6)	666 (16.4)
	≥ 200,000	1636	1227 (75.0)	257 (15.7)	152 (9.3)	1246 (76.2)	93 (5.7)	297 (18.1)
Family structure	Core family	14,028	11,086 (79.0)	1933 (13.8)	1009 (7.2)	10,968 (78.2)	910 (6.5)	2150 (15.3)
	Single parent	1288	1035 (80.3)	167 (13.0)	86 (6.7)	1031 (80.1)	107 (8.3)	150 (11.6)
	Live with custodians other than parents	459	362 (78.9)	65 (14.1)	32 (7.0)	347 (75.6)	42 (9.2)	70 (15.2)
	Extended family	4354	3402 (78.1)	619 (14.2)	333 (7.7)	3199 (73.5)	344 (7.9)	811 (18.6)
Smoking (active)	No	19,834	15,735 (79.3)	2713 (13.7)	1386 (7.0)	15,355 (77.4)	1358 (6.9)	3121 (15.7)
	Yes	295	150 (50.8)	71 (24.1)	74 (25.1)	190 (64.4)	45 (15.3)	60 (20.3)
Alcohol drinking	No	19,228	15,312 (79.6)	2596 (13.5)	1320 (6.9)	14,935 (77.7)	1316 (6.8)	2977 (15.5)
	Yes	901	573 (63.6)	188 (20.9)	140 (15.5)	610 (67.7)	87 (9.7)	204 (22.6)
Physical activity (min/week)	No	8471	6808 (80.4)	1095 (12.9)	568 (6.7)	6584 (77.7)	510 (6.0)	1377 (16.3)
	1 to 419	4251	3309 (77.8)	630 (14.8)	312 (7.3)	3268 (76.9)	344 (8.1)	639 (15.0)
	≥ 420	7407	5768 (77.9)	1059 (14.3)	580 (7.8)	5693 (76.9)	549 (7.4)	1165 (15.7)
ADHD	No	19,540	15,483 (79.2)	2675 (13.7)	1382 (7.1)	15,155 (77.5)	1361 (7.0)	3024 (15.5)
	Yes	589	402 (68.2)	109 (18.5)	78 (13.2)	390 (66.2)	42 (7.1)	157 (26.7)
History of asthma	No	19,826	15,652 (79.0)	2746 (13.8)	1428 (7.2)	15,317 (77.3)	1388 (7.0)	3121 (15.7)
	Yes	303	233 (76.9)	38 (12.5)	32 (10.6)	228 (75.2)	15 (5.0)	60 (19.8)
History of allergic rhinitis	No	17,857	14,245 (79.8)	2358 (13.2)	1254 (7.0)	13,881 (77.7)	1233 (6.9)	2743 (15.4)
	Yes	2272	1640 (72.2)	426 (18.7)	206 (9.1)	1664 (73.2)	170 (7.5)	438 (19.3)
History of eczema	No	19,404	15,366 (79.2)	2643 (13.6)	1395 (7.2)	15,043 (77.5)	1344 (6.9)	3017 (15.5)
	Yes	725	519 (71.6)	141 (19.5)	65 (9.0)	502 (69.2)	59 (8.1)	164 (22.6)

*ADHD* attention deficit/hyperactivity disorder.

The prevalence of tobacco use was 1.46%, while 21.1% of the participants reported SHS exposure for at least 1 day/week. The prevalence of ADHD determined by the validated scale was 2.93%. The prevalence rates of atopic dermatitis and hand eczema were 3.86% and 3.35%, respectively (Table 2), and interdigital eczema was the most common subclass of hand eczema (1.53%).

**Table 2:** Prevalence of atopic dermatitis and hand eczema among the participants.

Diseases and types	N (%)
Atopic dermatitis	776 (3.86)
**Hand eczema**	674 (3.35)
Interdigital eczema	317 (1.57)
Chronic fissured hand eczema	54 (0.27)
Recurrent vesicular hand eczema	220 (1.09)
Hyperkeratotic eczema	31 (0.15)
Nummular hand eczema	52 (0.26)

### Center effect of clinical diagnoses

Two study sites showed significant center effects, where the prevalence rates of atopic dermatitis and hand eczema were significantly lower than the other three sites (Supplementary Table S1). The ICCs for clinical diagnoses of atopic dermatitis and hand eczema were 19.3% and 15.2%, respectively. After excluding the data from the two study sites, the ICC was minimal (< 0.1%). As a result, random-intercept model was used in the further analysis.

### Effect of SHS exposure

**Figure 1:**
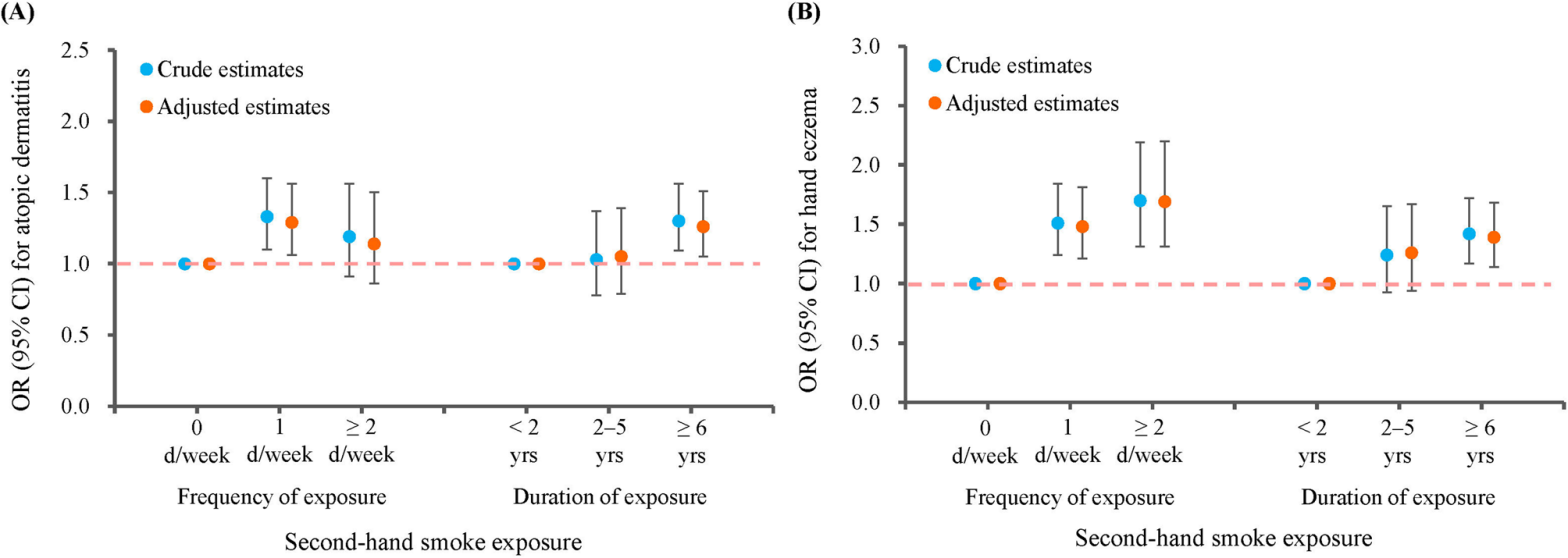
Association of second-hand smoke exposure on atopic dermatitis and hand eczema. (**A**) Atopic dermatitis. (**B**) Hand eczema.

Crude and adjusted estimates consistently showed that SHS exposure was significantly associated with higher risks of atopic dermatitis and hand eczema (Fig. 1). Both the frequency and duration of exposure were positively associated with hand eczema in a clear dose–response manner. In contrast, the frequency of exposure showed some variations in its correlation with atopic dermatitis, where exposure of 1 day/week was associated with the greatest risk (AOR = 1.29; 95% CI 1.06–1.56; *P* = 0.010) (Supplementary Table S2).

### Joint effect of the frequency and duration of SHS exposure

**Figure 2:**
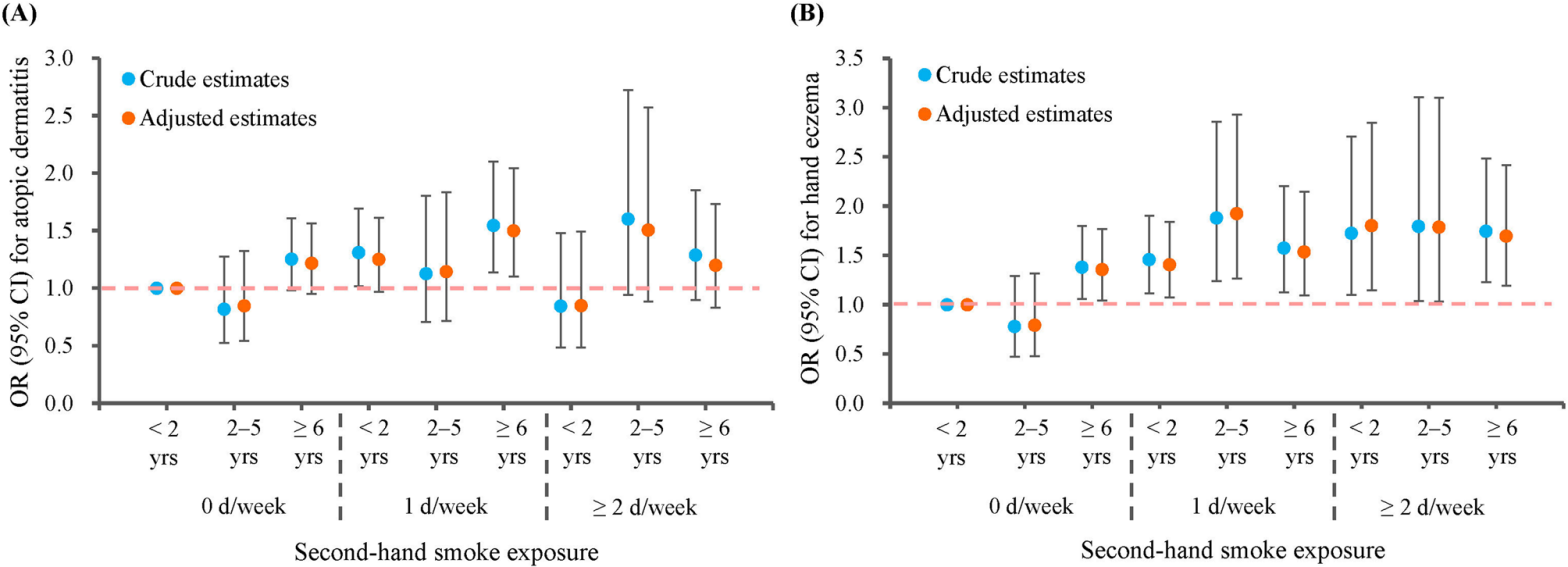
Joint effect of the frequency and duration of second-hand smoke exposure on atopic dermatitis and hand eczema (**A**) Atopic dermatitis. (**B**) Hand eczema.

Joint effect analysis showed that cumulative SHS exposure was associated with atopic dermatitis and hand eczema in a does-response manner with variations (Fig. 2). The risk of atopic dermatitis peaked when duration of exposure exceeded 6 years (AOR = 1.50; 95% CI 1.10–2.04; *P* = 0.010) or frequency of exposure exceeds 2 d/week (AOR = 1.51; 95% CI 0.88–2.57; *P* = 0.131) although statistically insignificant owing to limited sample size in some categories (Supplementary Table S3). The risk of hand eczema increased along with the cumulative dose of smoke exposure, but when the frequency of exposure exceeded 2 day/week, a longer duration did not further increase the risk.

### Effects of covariates and mediators

The associations of other covariates with atopic dermatitis and hand eczema are shown in supplementary Table S4. Active smoking was also associated with higher risk of atopic dermatitis (AOR = 1.51; 95% CI 0.80–2.85; *P* = 0.201) and hand eczema (AOR = 1.33; 95% CI 0.68–2.60; *P* = 0.399), although the associations were not statistically significant owing to the small number of active smokers (1.46%).

ADHD was significantly associated with hand eczema (AOR = 2.86; 95% CI 2.14–3.82; *P* < 0.001) but not atopic dermatitis (*P* = 0.663). ADHD significantly mediated a small proportion (6.17%) of SHS’s effect on hand eczema, but not on atopic dermatitis (Supplementary Table S5).

### Subgroup analysis for hand eczema

Considering the sample size of each subclass of hand eczema, we separately analyzed interdigital eczema and recurrent vesicular hand eczema, and combined chronic fissured hand eczema, hyperkeratotic eczema and nummular hand eczema as one group (Supplementary Table S6). Results remained consistent in general, even though interdigital and recurrent vesicular hand eczema were not significantly associated with the duration of exposure, while other types of hand eczema were not significantly associated with the frequency of exposure. Despite the variations in statistical significance, the observed effect size indicated a robust association of exposure with hand eczema.

### Sensitivity analysis

Sensitivity analysis was performed by excluding 5304 subjects from the two study sites (Urumqi and Hohhot) which showed significant center effect. The results were consistent with the full dataset analyses, although minor changes in effect size were observed (Supplementary Table S7).

## Discussion

Our study investigated the associations of self-reported SHS exposure with diagnosed atopic dermatitis and hand eczema in a group of first-year college students in China. The intensity of exposure, as measured by frequency and duration, was associated with both health outcomes in a does-response manner. The SHS-related risk for hand eczema was generally stronger than that for atopic dermatitis. ADHD mediated minimal or no effect of SHS on hand eczema and atopic dermatitis. Regarding the high homogeneity of our study population (almost the same birth cohort), we concluded that exposure to SHS during childhood and puberty is an independent risk factor for atopic skin diseases in adolescence.

The prevalence of current active smoking in our study was 1.46%, indicating that SHS exposure among adolescents is mainly attributable to the smoking behavior of their male family members rather than peers. This inference is in line with the result that participants from bigger families reported longer duration of exposure. However, the rate of tobacco use was lower than previous reports from multi-country surveys among students aged 12 to 15 (Chinese: 3.3% to 19.4%)^19–21^. The gap in the prevalence of tobacco use between our study and previous reports might be related to the selection bias among college students who had better performance and less risk behaviors during their high school period. This selection bias, however, enables us to investigate the association of SHS with health outcomes by almost excluding the effect of active smoking.

A previous meta-analysis showed that SHS was associated with a stronger effect on atopic dermatitis in adults (pooled OR = 3.62; 95% CI 1.71–7.69) than children (pooled OR = 1.15; 95% CI 1.01–1.30)^9^, while our study found a moderate effect size of 1.29 (95% CI 1.06–1.56) between the above values. This is possibly because of the delayed disease manifestation with a lifelong cumulative effect of smoke exposure, as indicated by a previous case–control study of adult-onset atopic dermatitis^22^. We also observed a stronger effect of SHS on atopic dermatitis among those who reported mild exposure (1 day/week) than extensive exposure (≥ 2 days/week). This is consistent with previous studies^9,23^, although the finding violates the hypothesis of dose–response relationship and the mechanism is not clear.

Several studies have identified SHS exposure as a risk factor for ADHD in children^15,24,25^. Hyperactivity increases the chance of exposure to irritants, contact allergens, and friction, and therefore may increase the risk of hand eczema in sensitized persons. This is similar with the finding that the association of SHS with hand eczema was stronger among occupational workers than the general population^10^. Except behavioral factors, ADHD is associated with atopic and allergic diseases according to population-based studies^26,27^, possibly through immunological mechanisms involving IgE hypersecretion, increased eosinophilic activity, and T helper 2 cytokine over-secretion^16^. Our finding, however, did not support the mediation effect of ADHD in the association linking SHS and eczema. This indicates that the SHS is a independent risk factor for atopic dermatitis and hand eczema. Nevertheless, health policy makers, especially in countries that have not yet implemented the smoke-free legislation, must keep in mind that SHS is a public health threat for non-smokers.

### Limitations

The primary limitation of the study is that no conclusion on causation can be drawn owing to the cross-sectional design. However, we retrospectively investigated the exposure to SHS and diagnosed the skin diseases at the timepoint of survey. It is reasonable to postulate that exposure to SHS mostly occurred prior to the development of skin diseases. Reversed causality is less likely, because skin disease may negatively impact health behaviors such as active smoking, but not SHS exposure. The second limitation is the lack of assessment for the severity of skin diseases owing to limited feasibility in a large-scale field survey. Third, family history of allergic diseases has not been included in covariates due to potential recall bias. Genetic factors might have an impact on incidence and severity of atopic dermatitis after smoke exposure and further researches are needed to assess the impact. The Forth limitation is the presence of center effect of clinical diagnosis, partly owing to the difference in the detection rate by dermatologists. Nevertheless, we used mixed models with random intercepts to deal with this ubiquitous problem in studies under a cluster sampling frame, and the sensitivity analysis showed consistent results. Last but not least, the effects of indoor and outdoor air pollution in addition to SHS were not evaluated in our study, while a recent study among children indicated some synergistic effects between these exposures^28^. The strengths of the study include: the accuracy of diagnoses as determined by dermatologists; relatively large sample size that suffices power to detect differences; homogeneity of the study population; and robustness of the statistical model that is capable of dealing with intracluster correlation and generating unbiased estimations.

## Conclusion

Our study identified a dose–response association of previous exposure to SHS with atopic dermatitis and hand eczema in late adolescence in a group of college students in China. The association was independent from ADHD that linked both SHS and eczema. The finding implicates that adult family members should avoid smoking in the presence of their children, in order to protect their children from atopic skin diseases and other health risks.

## Supplementary information

Supplementary Information.

## Data Availability

The data and materials generated or analyzed during this study are provided as the Supplementary Information Files.
