# Supplementary material for: Associations of second-hand smoke exposure with hand eczema and atopic dermatitis among college students in China
Source: Sci Rep. 2020 Oct 15;10:17400. doi: 10.1038/s41598-020-74501-2 (PMC7562873; doi:10.1038/s41598-020-74501-2)
Supplement: Supplementary file 1 — Supplementary Information. [file 41598_2020_74501_MOESM1_ESM.pdf]

Supplementary Information

Supplementary Figure S1. Study sites and geographic distribution of the student's hometown. (A) Study sites. (B) Student's hometown province.

Supplementary Table S1. Center effect of clinical diagnoses

Supplementary Table S2. Association of second-hand smoke exposure on atopic dermatitis and hand eczema

Supplementary Table S3. Joint effect of the frequency and duration of second-hand smoke exposure on atopic dermatitis and hand eczema

Supplementary Table S4. Full model: associations of other covariates with atopic dermatitis and hand eczema

Supplementary Table S5. Mediation effect analysis for attention deficit / hyperactivity disorder

Supplementary Table S6. Subgroup analysis by subclasses of hand eczema

Supplementary Table S7. Sensitivity analysis by excluding 5304 observations from two study sites with center effect

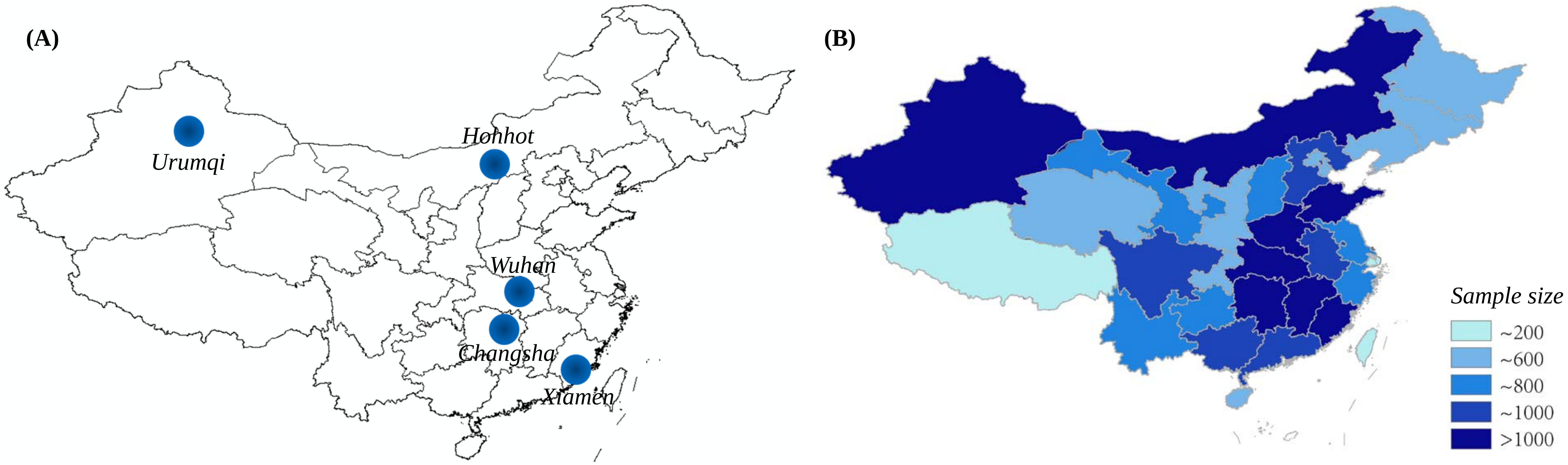

Supplementary Figure S1. Study sites and geographic distribution of the student's hometown.

(A) Study sites. (B) Student's hometown province.

Supplementary Table S1. Center effect of clinical diagnoses

Center	Atopic dermatitis	Hand eczema	Center effect
Changsha	212 (4.2)	185 (3.7)	No
Wuhan	276 (4.9)	218 (3.9)	No
Xiamen	184 (4.4)	183 (4.3)	No
Urumqi	33 (1.1)	49 (1.7)	Yes
Hohhot	71 (3.0)	39 (1.6)	Yes
ICC (%) in full dataset	19.3	15.2	Yes
ICC (%) after excluding sites with center effect	0.1	0.0	No

ICC: intracluster correlation coefficient.

Supplementary Table S2. Association of second-hand smoke exposure and atopic dermatitis and hand eczema

	Atopic dermatitis				Hand eczema			
Second-hand smoke exposure	N (%)	OR (95% CI)	AOR (95% CI) [a]	*P*	N (%)	OR (95% CI)	AOR (95% CI) [a]	*P*
Frequency of exposure (d/week)								
0	575 (3.6)	1 (reference)	1 (reference)		472 (3.0)	1 (reference)	1 (reference)	
1	140 (5.0)	1.33 (1.10–1.60)	1.29 (1.06–1.56)	0.010	131 (4.7)	1.51 (1.24–1.84)	1.48 (1.21–1.81)	<0.001
≥ 2	61 (4.2)	1.19 (0.91–1.56)	1.14 (0.86–1.50)	0.361	71 (4.9)	1.70 (1.31–2.19)	1.69 (1.31–2.20)	<0.001
Duration of exposure (years)								
< 2	563 (3.6)	1 (reference)	1 (reference)		475 (3.1)	1 (reference)	1 (reference)	
2–5	55 (3.9)	1.03 (0.78–1.37)	1.05 (0.79–1.39)	0.751	55 (3.9)	1.24 (0.93–1.65)	1.26 (0.94–1.67)	0.120
≥ 6	158 (5.0)	1.30 (1.09–1.56)	1.26 (1.05–1.51)	0.014	144 (4.5)	1.42 (1.17–1.72)	1.39 (1.14–1.68)	<0.001

OR: odds ratio. AOR: adjusted odds ratio. CI: confidence interval.

[a] Adjusted for the random effect of university and fixed effect of age, gender, ethnicity, family income, family structure, active smoking, alcohol drinking, physical activity, attention deficit / hyperactivity disorder, asthma, and allergic rhinitis.

Supplementary Table S3. Joint effect of frequency and duration of second-hand smoke exposure on atopic dermatitis and hand eczema

Frequency	Duration	Atopic dermatitis				Hand eczema			
(d/week)	(years)	N (%)	OR (95% CI)	AOR (95% CI) [a]	*P*	N (%)	OR (95% CI)	AOR (95% CI) [a]	*P*
0	< 2	477 (3.5)	1 (reference)	1 (reference)		388 (2.9)	1 (reference)	1 (reference)	
0	2–5	21 (3.1)	0.82 (0.52–1.27)	0.85 (0.54–1.32)	0.467	16 (2.4)	0.78 (0.47–1.29)	0.79 (0.48–1.32)	0.371
0	≥ 6	77 (4.7)	1.25 (0.98–1.61)	1.22 (0.95–1.56)	0.121	68 (4.2)	1.38 (1.06–1.80)	1.36 (1.04–1.77)	0.025
1	< 2	73 (4.8)	1.31 (1.02–1.69)	1.25 (0.97–1.61)	0.086	66 (4.4)	1.46 (1.11–1.90)	1.41 (1.07–1.84)	0.013
1	2–5	19 (4.3)	1.13 (0.70–1.80)	1.14 (0.71–1.83)	0.577	25 (5.7)	1.88 (1.24–2.85)	1.92 (1.26–2.93)	0.002
1	≥ 6	488 (5.8)	1.55 (1.14–2.10)	1.50 (1.10–2.04)	0.010	40 (4.8)	1.57 (1.13–2.20)	1.53 (1.10–2.15)	0.013
≥ 2	< 2	13 (2.9)	0.84 (0.48–1.48)	0.85 (0.48–1.49)	0.569	21 (4.7)	1.73 (1.10–2.71)	1.80 (1.14–2.84)	0.011
≥ 2	2–5	15 (5.2)	1.60 (0.94–2.72)	1.51 (0.88–2.57)	0.133	14 (4.8)	1.79 (1.04–3.10)	1.79 (1.03–3.10)	0.039
≥ 2	≥ 6	33 (4.6)	1.29 (0.90–1.85)	1.20 (0.83–1.73)	0.330	36 (5.0)	1.75 (1.23–2.48)	1.70 (1.19–2.42)	0.004

OR: odds ratio. AOR: adjusted odds ratio. CI: confidence interval.

[a] Adjusted for the random effect of university and fixed effect of region, age, gender, ethnicity, family income, family structure, active smoking, alcohol drinking, physical activity, attention deficit / hyperactivity disorder, asthma, and allergic rhinitis.

Supplementary Table S4. Full model for the associations of other covariates with atopic dermatitis and hand eczema

	Atopic dermatitis		Hand eczema	
Covariates	AOR (95% CI)	*P*	AOR (95% CI)	*P*
Region of hometown				
North	1 (reference)		1 (reference)	
Northeast	1.24 (0.81–1.89)	0.326	1.30 (0.78–2.18)	0.310
East	0.92 (0.70–1.22)	0.567	1.40 (1.00–1.97)	0.051
Central	1.12 (0.85–1.48)	0.426	1.53 (1.09–2.15)	0.014
South	1.34 (0.96–1.85)	0.084	1.73 (1.17–2.54)	0.006
Southwest	1.21 (0.88–1.67)	0.243	1.47 (1.00–2.15)	0.048
West	0.68 (0.47–1.00)	0.047	0.73 (0.49–1.10)	0.132
Age (years)	0.96 (0.86–1.08)	0.506	0.96 (0.86–1.08)	0.524
Female gender	1.43 (1.23–1.67)	<0.001	1.39 (1.18–1.64)	<0.001
Han ethnicity	1.08 (0.86–1.36)	0.493	0.95 (0.75–1.20)	0.650
Family income		.		
< 10,000	1 (reference)		1 (reference)	
10,001 to 30,000	1.25 (0.89–1.76)	0.198	0.88 (0.65–1.18)	0.385
30,001 to 50,000	1.53 (1.09–2.16)	0.014	0.79 (0.58–1.08)	0.142
50,001 to 99,999	1.48 (1.06–2.07)	0.021	0.84 (0.62–1.13)	0.246
100,000 to 199,999	1.57 (1.12–2.19)	0.009	0.70 (0.51–0.95)	0.022
≥ 200,000	1.82 (1.26–2.63)	0.002	0.77 (0.53–1.10)	0.147
Family structure				
Core family	1 (reference)		1 (reference)	
Single parent	1.10 (0.81–1.48)	0.561	0.93 (0.67–1.30)	0.686
Live with grandparents	1.10 (0.69–1.75)	0.687	1.56 (1.03–2.36)	0.034
Extended family	0.93 (0.78–1.11)	0.431	0.96 (0.79–1.16)	0.675
Active smoking	1.51 (0.80–2.85)	0.201	1.33 (0.68–2.60)	0.399

Alcohol drinking	0.86 (0.59–1.27)	0.456	0.96 (0.64–1.43)	0.837
Physical activity (min/week)				
No	1 (reference)		1 (reference)	
1 to 419	1.18 (0.98–1.43)	0.085	0.88 (0.71–1.09)	0.251
≥ 420	1.00 (0.85–1.19)	0.971	0.99 (0.82–1.18)	0.877
ADHD	0.91 (0.59–1.40)	0.663	2.86 (2.14–3.82)	<0.001
Asthma	2.69 (1.85–3.91)	<0.001	1.88 (1.18–2.99)	0.008
Allergic rhinitis	1.50 (1.23–1.82)	<0.001	1.31 (1.05–1.63)	0.017

AOR: adjusted odds ratio. CI: confidence interval. ADHD: attention deficit / hyperactivity disorder.

Supplementary Table S5. Mediation effect analysis for attention deficit / hyperactivity disorder

Outcome	Total effect of SHS	Direct effect of SHS	Mediation effect of ADHD	% mediated	*P* for mediation effect
Atopic dermatitis	0.011	0.011	$–2.76×10^{–5}$	–0.24	0.880
Hand eczema	0.014	0.013	$8.67×10^{–4}$	6.17	<0.001

SHS: second-hand smoke. ADHD: attention deficit / hyperactivity disorder

Supplementary Table S6. Subgroup analysis by subclasses of hand eczema

	Interdigital eczema			Recurrent vesicular hand eczema			Other types [b]		
Second-hand smoke exposure	N (%)	AOR (95% CI) [a]	*P*	N (%)	AOR (95% CI) [a]	*P*	N (%)	AOR (95% CI) [a]	*P*
Frequency of exposure (d/week)									
0	218 (1.4)	1 (reference)		155 (1.0)	1 (reference)		99 (0.6)	1 (reference)	
1	68 (2.5)	1.76 (1.32–2.31)	<0.001	39 (1.5)	1.30 (0.91–1.86)	0.146	24 (0.9)	1.26 (0.80–1.97)	0.323
≥ 2	31 (2.2)	1.66 (1.13–2.44)	0.010	26 (1.8)	1.98 (1.29–3.02)	0.002	14 (1.0)	1.32 (0.75–2.35)	0.339
Duration of exposure (years)									
< 2	229 (1.5)	1 (reference)		156 (1.0)	1 (reference)		90 (0.6)	1 (reference)	
2–5	25 (1.8)	1.24 (0.81–1.88)	0.319	19 (1.4)	1.32 (0.81–2.13)	0.265	11 (0.8)	1.25 (0.67–2.35)	0.487
≥ 6	63 (2.0)	1.29 (0.97–1.72)	0.077	45 (1.5)	1.31 (0.93–1.83)	0.119	36 (1.2)	1.81 (1.23–2.69)	0.003

OR: odds ratio. AOR: adjusted odds ratio. CI: confidence interval.

[a] Adjusted for the random effect of university and fixed effect of age, gender, ethnicity, family income, family structure, active smoking, alcohol drinking, physical activity, attention deficit / hyperactivity disorder, asthma, and allergic rhinitis.

[b] Other types include chronic fissured hand eczema, hyperkeratotic eczema, and nummular hand eczema.

Supplementary Table S7. Sensitivity analysis by excluding 5304 observations from two study sites with center effect

	Atopic dermatitis				Hand eczema			
Second-hand smoke exposure	N (%)	OR (95% CI)	AOR (95% CI) [a]	*P*	N (%)	OR (95% CI)	AOR (95% CI) [a]	*P*
Frequency of exposure (d/week)								
0	491 (4.3)	1 (reference)	1 (reference)		403 (3.5)	1 (reference)	1 (reference)	
1	127 (5.5)	1.31 (1.07–1.60)	1.28 (1.04–1.56)	0.018	121 (5.3)	1.53 (1.24–1.89)	1.49 (1.21–1.84)	<0.001
≥ 2	54 (5.2)	1.24 (0.93–1.65)	1.18 (0.88–1.58)	0.267	62 (6.0)	1.75 (1.33–2.31)	1.75 (1.33–2.32)	<0.001
Duration of exposure (years)								
< 2	481 (4.3)	1 (reference)	1 (reference)		406 (3.6)	1 (reference)	1 (reference)	
2–5	50 (4.5)	1.05 (0.78–1.41)	1.07 (0.79–1.44)	0.668	49 (4.4)	1.23 (0.91–1.66)	1.25 (0.92–1.70)	0.151
≥ 6	141 (5.5)	1.28 (1.06–1.55)	1.24 (1.02–1.51)	0.030	131 (5.1)	1.41 (1.15–1.73)	1.37 (1.12–1.68)	0.002

OR: odds ratio. AOR: adjusted odds ratio. CI: confidence interval.

[a] Adjusted for the random effect of university and fixed effect of age, gender, ethnicity, family income, family structure, active smoking, alcohol drinking, physical activity, attention deficit / hyperactivity disorder, asthma, and allergic rhinitis.
